# Studying asphyxiation in the lab: the role of experimental evidence in cause-of-death inquiry

**DOI:** 10.1007/s40656-025-00712-3

**Published:** 2026-01-19

**Authors:** Enno Fischer, Saana Jukola

**Affiliations:** 1 Dresden University of Technology, Zellescher Weg 17, 01069 Dresden, Germany; 2 University of Twente, Drienerlolaan 5, 7522 NB Enschede, The Netherlands; 3 Center for Philosophy of Epidemiology, Medicine, and Public Health, University of Johannesburg, Johannesburg, South Africa

**Keywords:** Evidence, Experiment, Causation, Forensic medicine, Asphyxiation, Excited delirium syndrome

## Abstract

Like most scientific and medical disciplines, forensic medicine employs evidence from experimental studies. Yet, unlike most disciplines, forensic medicine is primarily interested in the post-hoc evaluation of individual causal claims. How does experimental work that is performed under laboratory conditions bear on the assessment of field cases? We argue that experimental studies in forensic medicine help identify or exclude *potential* causes of death. Potential causes will not explain why an individual died. Yet they can be important to rebut claims to the impossibility of a certain course of events. We support our argument by looking at experimental studies of asphyxiation. These studies have been central to recent academic and public debate of death-in-custody. While some take the studies to show that restraint positions employed by law enforcement can cause death, others dispute this. We analyze the causal claims put forward by experimental asphyxiation studies and show that some attempts to disprove the risks associated with restraint positions involve ‘false advertising’: a mismatch between the study’s methodology and its purported goals.

## Introduction

Forensic medicine, like other forensic disciplines, plays an important role in deciding legal cases. Yet the reliability of evidential practices in forensics has been a matter of intense debate in recent decades (National Research Council, 2009; PCAST, 2016; Weyermann & Roux, 2021). In particular, it is well-recognized that forensic medicine lacks a systematic methodology for causal analysis (Meilia et al., 2020). This calls for an epistemology of forensic medicine and, in particular, an analysis of the evidential practices in cause-of-death inquiry.

Here we contribute to this goal by looking at the role of experimental evidence in cause-of-death inquiry.^1^ Like most scientific and medical disciplines, forensic medicine employs evidence from experimental studies. Yet, unlike most disciplines, forensic medicine is primarily interested in the post-hoc evaluation of individual causal claims. This raises questions: What is the role of experiments in forensic medicine, and, more specifically, how does experimental work that is performed under strongly controlled laboratory conditions bear on the assessment of field cases?

More precisely, we argue that an important role played by experimental studies in forensic medicine is to help identify or exclude *potential* causes of death. The aim of employing forensic medical expertise is to determine responsibility in legal processes. This ultimately requires judgements of what *actually* caused an individual’s being harmed. Evidence for such claims of actual causation, however, requires assessing claims of potential causation, that is, the question whether a certain factor can at all bring about the harm in question. A claim of potential causation will not necessarily explain why an individual died. Yet it can be important to rebut claims to the impossibility of a certain course of events. Thus, claims of potential causation help identify the hypotheses that are worthy of further pursuit when it comes to searching for the actual causes of an individual’s death.

Moreover, we show that experimentally establishing claims of potential causation comes with a number of specific challenges. In particular, we show that there is an asymmetry between ‘can cause’ claims and ‘cannot cause’ claims that concerns both the efforts required to establish such claims and the evidential consequences of such claims. This affects how such results are to be communicated: in communicating results of experimental studies, one should be particularly cautious to avoid “false advertising” (Carrier, 2013): studies should not give the impression that they are addressing causal questions that they in fact bypass.

Our main example are studies concerned with the causal role of positional restraint in cases of death-in-custody. We focus on these studies not only because they help to highlight the philosophical argument we want to make. We have chosen the example also because of its societal relevance. The use of restraint techniques by law enforcement officers has been controversial because of potential risks of restraint or positional asphyxia. An example is the so-called ‘prone maximal restraint position’ (PMRP) which involves being placed prone with the wrists and ankles bound behind the back. There has been fierce public and academic debate about the effect of restraint techniques. On the one hand, there are case reports and experiments that draw a connection between sudden death and the use of restraint techniques (e.g., Reay et al., 1988, 1992; O’Halloran & Frank, 2000). On the other hand, some experimental studies (e.g., Chan et al., 2004) have been taken to indicate that the restraint position does not play an important role in explaining deaths.

Explaining sudden death in police custody is a high-stakes question as recent discussions of the so-called “Excited Delirium Syndrome” (ExDS) indicate. While some see ExDS as a genuine condition and potential cause of death, others have argued that it is a made-up condition to explain away asphyxiation deaths that are ultimately caused by excessive police violence (for an overview see, e.g., Fischer & Jukola, 2024). More precisely, ExDS is sometimes argued to be the only available explanation of certain instances of death-in-custody. This line of argument rests heavily on excluding death by asphyxiation. Our analysis shows the methodological difficulties associated with attempts to experimentally show that restraint positions ‘cannot’ cause death-in-custody and it highlights the associated risks of false advertising of evidential claims in forensic medicine.

The argument proceeds as follows. In Sect. 2 we provide some background on evidential practices in forensic medicine. In Sect. 3 we have a closer look at the kinds of causal claims that are at stake in experimental studies in forensic medicine, and we argue that they can be characterized as claims of potential causation. We also provide a preliminary characterization of such claims and discuss how they are to be evaluated. In Sects. 4 and 5, these claims will be supported and extended by a detailed look at the main experimental studies concerned with the effects of restraint positions and their relation to asphyxiation. We will show that there is an asymmetry between ‘can cause’ claims and ‘cannot cause’ claims that concerns both the efforts required to establish such claims and their evidential consequences. In Sect. 6 we will highlight problems of ‘false advertising’ that follow from this asymmetry.

## Evidential practice in forensic medicine

Philosophical discussion about the use of evidence in medicine has burgeoned in the last decades (e.g., Worrall, 2010; Howick, 2011; Parkkinen et al., 2018; for a recent overview see Holman & Sung, 2025). This literature has focused on medical research and practice ultimately aiming at diagnosing, caring, or curing disease. As we show in this section, evidential practices in forensic medicine have some salient differences to those central in the clinical setting and mostly discussed in philosophy of medicine.

Forensic medicine is a medical subfield whose primary purpose is to provide evidence in legal processes instead of diagnosing or healing individuals.The duties of forensic medical experts differ between jurisdictions and countries: while in some contexts they perform examinations also on living clients (e.g., in cases of suspected sexual abuse), in other jurisdictions they only investigate the dead. Here we will be concerned with the latter cases. In particular, we will be concerned with evidential practices in the context of finding a cause of death in cases where an individual has passed under unclear circumstances.

Before we proceed to discuss the challenges of producing and applying evidence in forensic medicine, it is important to make a quick note about the terminology: The cause of death is “the disease or injury that initiated the train of morbid events leading directly to death, or the circumstances of the accident or violence which produced the fatal injury” (World Health Organization, 2022, Sect. 04). For example, a cause of death can be strangulation, alcohol abuse, or lung cancer. The cause of death is to be distinguished from the manner of death, which is primarily a legal category. In the United States (U.S.), for example, manners of death can be classified as natural, accident, suicide, homicide, and undetermined. It is then possible that two individuals have the same cause of death, but the manner of their deaths differ.

As the debate on the COVID-19 death rate determination demonstrates, determining the cause of a death can become a controversial matter even in circumstances where no foul play is suspected (e.g., Amoretti & Lalumera, 2021). Especially in cases of comorbidities, how the real, underlying cause of death is determined can depend on contextual factors. When a suicide, homicide or terminal occupational illness is a possibility, causal inferences related to past events and how they may have affected the death become even more contested. According to Madea and Rothschild (2010), the scientific quality of cause and manner of death determination has been debated and it has been claimed that postmortem investigations fail to recognize all unnatural deaths.

The contested nature of cause of death determination is related to both evidential practices in forensic medicine and the nature of the phenomena of interest. Many procedures used in determining the cause of death are not standardized, and the professional judgment of the forensic medical expert is central in determining what techniques are used in the examination. According to Meilia et al. (2018), procedures vary between centers, many experts rely on experience and use different methods in formulating expert opinions, which can give rise to conflicting conclusions about the cause or manner of death. Consequently, deaths can be classified differently even by experts with similar backgrounds (Timmermans, 2007). Many authors have argued that causal inferences in forensic medicine are woefully subjective in nature (Meilia et al., 2018, 2020; for a discussion of cognitive bias in forensic medicine, see Dror et al., 2021).

A challenge in forensic medical practice is establishing what the evidence is and how it should be interpreted. The central source of evidence is autopsy, in which the body and its organs are inspected in order to observe abnormalities (Timmermans, 2007; Saukko & Pollak, 2009). Data of the body and its different organs is recorded, injuries are described, and the postmortem investigation also involves collecting samples for histological and toxicological analyses (Dettmeyer et al., 2014). In some cases, additional samples of tissue need to be collected. Findings are recorded in the autopsy report, which should also include information about negative findings (ibid.).

In the forensic medicine community, it is generally acknowledged that physical evidence from autopsy and the results from analyses do not always lead to a conclusion about the cause and manner of death (Meilia et al., 2020). Sometimes this is due to the advanced decomposition of the body, or the extensive nature of injuries, but even in less complicated cases the autopsy data has to be interpreted on the basis of other available knowledge. As Timmermans (2007, p. 67) notes, “[t]he autopsy report reads like a straightforward descriptive medical file, but what appears and what is left out—even if it constitutes a positive finding—depends upon the circumstances of the death, the history, and the scene investigation”. For instance, the medical history of the deceased individual and the circumstances of their death are relevant for determining whether someone died by natural causes or external influence. It is recognized that this is particularly difficult in cases of death in custody, which will be discussed in what follows (Mitchell Jr. & Aronson, 2023). In addition to such contextual factors, existing pathophysiological knowledge and knowledge from other specialties in and outside of medicine need to be considered before conclusions about the cause of death can be drawn. For example, epidemiological evidence about the prevalence of cot deaths is relevant for determining the cause of death of a young infant (Freeman et al., 2008).

What is important for our argument here, is the relevance that case studies and experiments have for interpreting the outcomes of autopsy and reaching conclusions about the cause of death. A considerable part of publications in forensic medicine have been case studies (Madea, 2007). The prevalent role of this study type is in tension with the principles of evidence-based medicine (EBM), which guide most clinical practice today. In EBM, different types of evidence are ranked in the so-called evidence hierarchy (Howick, 2011). In this hierarchy, meta-analyses and systematic reviews of RCTs are considered to provide the most reliable evidence, followed by RCTs, and observational studies. Mechanistic evidence, including pathophysiological evidence is ranked close to the bottom, together with case studies and expert judgment. The reason for the low ranking of case reports and case series is that they are potentially anecdotal: the reported phenomenon may be a coincidence, and the description can be influenced by different biases (Nissen & Wynn, 2014). Because of this, a report of, say, the progression of a disease in a patient or treatment success may not be generalizable to other cases. However, it has been acknowledged that case reports and series have an important function in generating new hypotheses and in the identification of previously undetected diseases or side effects (e.g., Vandenbroucke, 2001; Osimani, 2013). They can also provide more detailed accounts of events, which can inform further studies (e.g., Ankeny, 2014).

Philosophers of medicine have actively debated the evidence hierarchy, whether different study types can be ranked, and the foundations of EBM in general (e.g., Solomon, 2011; Stegenga, 2014; Rocca, 2018). In particular, methodological benefits and challenges of RCTs have attracted a lot of attention (e.g., Osimani, 2013, Fuller, 2019). Evidential pluralists and the proponents of the so-called EBM + movement have argued that mechanistic evidence in addition to evidence provided by RCTs is needed for making causal claims in clinical medicine (e.g., Parkkinen et al., 2018). When discussing the limitations of EBM principles in forensic medicine, it is important to note that, unlike in clinical medicine, the search for causes is always done retrospectively (Meilia et al., 2020). RCTs and many other study types are limited for obvious practical and ethical concerns (Meilia, 2022; see Jukola, 2019 for a discussion of similar issues in nutrition science). This means that the type of evidence EBM gives prominence to is not available for forensic medicine experts.

Forensic medicine experts utilize experiments that test hypotheses indirectly, however. Crash tests using dummies provide evidence that can be applied in the assessment of deaths and injuries involving vehicles (Liptai, 2007). Ballistic experiments on, e.g., ballistic gelatin, simulators or animal carcasses can be used for producing evidence for assessing gunshot wounds (Jussila, 2004). Animal experiments can be used also for assessing morphological changes after injuries and in toxicology (Madea et al., 2007; see Mole & Heyns, 2019 for a discussion of ethical issues related to the use of animals in forensic studies). Yet establishing external validity of such experiments is a challenge: are the outcomes of experiments conducted on animal carcasses or in a laboratory applicable to the assessment of actual cases in forensic medicine?^2^ What kind of assumptions concerning the similarities and differences between the experimental context and the context of the death of an individual have to be made before a forensic medical expert can use the evidence to draw conclusions? In the next sections, we address these questions and focus on the evidential role that experiments have in establishing claims of potential causation. While our focus will be a set of experiments conducted to establish causal claims about *asphyxiation*, we take these to support claims about more general features of evidential reasoning with experiments in forensic medicine (see Sect. 7 for additional discussion).

## Potential causation

Forensic medicine is primarily interested in the assessment of individual cause-of-death claims. How does experimental work under highly controlled laboratory conditions bear on such claims? Our main argument here is that experimental studies achieve this primarily through probing claims of *potential causation*. In this section we will provide some preliminary evidence for this hypothesis and make some necessary clarifications (What are such claims of potential causation? How are such claims to be evaluated?) before we turn to a more detailed look at the example of asphyxia studies in the following section.

The primary distinction here is that between potential causation and actual causation. An actual cause is an event or factor that does or did bring about a particular effect, as in the “bullet’s hitting the victim caused the victim’s death”.^3^ Potential causation, by contrast, is concerned with the question whether an event or factor *can* cause another event or factor.^4^ The question is whether under certain circumstances the factor can make a difference to the effect. Now, what “can make a difference” means is highly dependent on the scope of circumstances that we consider.

More precisely, one should distinguish (1) the range of the cause variable and (2) the relevant background conditions. (1) Range is important because a variable may be a difference-maker to another variable for some range of values, but not for others. Consider water consumption. Within certain bounds it will not make a difference to an individual’s survival how much water they drink. Yet in the extreme cases of dehydration or water intoxication it does make a difference to survival. Whether water consumption can make a difference depends on the specific quantities we are looking at. (2) Background conditions also play an important role. A variable may be a difference-maker to another variable under some background conditions but not under others. For example, if the water is contaminated or if the subject has problems metabolizing it, then consuming more or less of it may have an impact on the subject’s health and survival. Thus, when addressing a claim of potential causation, it is crucial to identify the relevant variable ranges and background conditions.

This makes the evaluation of claims of potential causation context sensitive. One needs to specify the variable ranges and background conditions that are relevant to the specific context under consideration.^5^ This has also consequences for the evaluation of experimental claims about potential causation. Experimental studies will test claims of potential causation for certain variable ranges and under specific and controlled background conditions. The results of the experimental study are informative about the target phenomenon only insofar as they allow inferences regarding the variable ranges and background conditions met in the field setting.

To make the implications of employing the actual/potential distinction clearer let us contrast the approach with extant work on causal reasoning. Common taxonomies of causal claims distinguish between generic causal claims (causal generalizations, causal laws) and claims of singular causation (also sometimes called actual causation). Generic causal claims describe regularities between kinds of events or factors, as in “smoking causes lung cancer”. Singular causation describes individual instances of causation, as in “Peter’s lung cancer was caused by smoking”.

Russo and Williamson (2011) employ such a taxonomy to examine causal reasoning in forensic autopsy. They argue that the primary mode of reasoning in this context is from generic causal claims to single-case causality. Generic causal claims like “striking matches is a cause of their lighting” are combined with single-case non-causal information such as the fact that a match has been struck to explain why the match is lit.

According to the generic-to-individual model, the goal of experiments would be to provide generic causal claims that can be applied in the assessment of individual cases of death. Yet, the goal of forensic claims derived from experiments is typically much more modest than suggested by the match-striking example. In the match-striking example the generic causal relation together with the fact that the match was struck strongly suggests a causal inference to the fact that the match is being lit. In the examples typically addressed by experiments in forensic medicine, however, the inferential connection is much less direct. As we will see below, the role of the asphyxia studies is not primarily to show that in certain instances restraint-induced asphyxia is the cause of death, but the research rather concerns whether the kinds of restraint applied in the field setting can at all lead to asphyxiation.

Claims of potential causation resemble “how-possibly explanations”. Dray (1963, 1964, 1968) has argued that how-possibly explanations represent a distinct mode of scientific explanation. Unlike Hempel-Oppenheim-style (1948) covering law explanations, they do not provide a basis for nomologically deducing the explanandum but only refer to necessary conditions of the explanandum phenomenon. These kinds of explanations respond to questions of “*how it could be* that a certain thing happened” (Dray, 1968, p. 390, emph. original). There has been an extensive debate about whether and under what circumstances how-possibly explanations are in fact a distinct mode of explanation and whether they have genuine explanatory value (see Reiner (1993) and Reinhard (2025) for overviews).

Here it is not so important whether claims of potential causation are the basis of a distinct mode of explanation. What matters is that like how-possibly explanations, claims of potential causation are particularly relevant when it comes to rebutting considerations of presumed impossibility. A claim of potential causation will not necessarily explain why an individual died. Yet it can be important to rebut claims to the impossibility of a certain course of events. In what follows we will see that this mode of causal reasoning is particularly important in the context of death by asphyxiation and associated discussions of the ExDS.

Moreover, it will be useful to delineate the current concept of potential causation from extant concepts of mechanistic *evidence* and mechanistic *explanation*. Mechanistic *evidence* is evidence of the presence of mechanisms such as involving “chemical reactions, electrical signals, alterations at the cellular level, etc.” (Russo & Williamson, 2007, p. 162). In the EBM + movement it has been argued that this kind of evidence needs to be combined with evidence of probabilistic dependencies to establish a causal claim in the health sciences (Russo & Williamson, 2007; Illari, 2011; Parkkinen et al., 2018; Wilde & Parkkinen, 2019). There are senses in which evidence of potential causation is both weaker and stronger than mechanistic evidence. Evidence of potential causation is weaker because it is not evidence to the effect that the factor in question is operative in general or even in concrete instances but merely evidence to the effect that the factor—if active—can bring about the effect. Evidence of potential causation is stronger than mechanistic evidence because it is evidence to the effect that the factor can make a difference to the outcome without necessarily being combined with additional evidence of probabilistic dependencies.

Potential causation also differs from mechanistic *explanation*. According to Machamer et al. (2000), mechanisms “are entities and activities organized such that they are productive of regular changes from start or set-up to finish or termination conditions” (3). Such mechanisms explain by “revealing the *productive* relation” that those entities and activities have with the mechanism’s end stage (22, emph. orig.). Moreover, “[m]echanism descriptions show *how possibly*, *how plausibly*, or *how actually* things work” (21, emph. orig.). Mechanistic explanations thus may act as evidential support for claims of potential causation. Evidence of potential causation, however, does not require mechanistic explanation. As we will see as we go on, discussions of death through asphyxiation remain relatively independent of concrete mechanisms. While theorizing about potential mechanisms of asphyxiation does play a role in the studies-to-be-discussed, the focus lies on the difference that restraint positions make to a series of parameters such as heart rate and blood oxygenation–but not the exact entities and activities that produce a low heart rate and oxygenation.

Instead, the claims of potential causation discussed here have similarities with claims of “causal competence” that result from experiments in disciplines such as paleobiology and archeology (Novick et al., 2020). These claims are far from providing decisive evidence of what happened in the past. Yet they help counter certain impossibility claims, e.g., the claim that it is impossible that ancient Peruvians settled in Rapa Nui (Easter Island) that was challenged by reenacting travels to Rapa Nui on rafts using ancient Peruvian technology.

Accordingly, the function of experiment in forensic medicine resembles that of experiment in disciplines concerned with the deep past, that is, disciplines that are concerned with explaining past events and that have very limited or indirect empirical access to its subject matter (evidence of the deep past is elusive, just as is evidence of the kinds of cases addressed in asphyxia studies). More precisely, forensic medicine is mostly concerned with what Novick et al. call specific projections, that is, “projecting from the processes observed in the lab to a specific instance of their operation” (ibid., 229)—as opposed to intermediate or general projections that work with limited or no such attributions.

An important evidential function of such claims concerns shutting down or opening lines of inquiry. If it is shown that a specific factor cannot be a cause of the effect under consideration (or does not have the causal competence to bring it about), then further inquiry into the operation of that factor will be discouraged. If it is shown that a factor can cause the effect (or does have the causal competence to bring it about), then further inquiry may well be pursuitworthy.

## Experimental asphyxia studies

In what follows we will discuss a series of experimental studies that address the causal role of body position and restraint in cases of death-in-custody. Looking at these examples will illustrate and support the claims about potential causation made in the foregoing section. Moreover, the studies are of interest because they highlight some of the challenges related to cause-of-death inquiry in a particularly clear way.

It should be emphasized that the goal here is not to provide a comprehensive review of the literature (see Steinberg (2021) for a recent example). Neither do we aim to assess or evaluate the performed studies at this stage. Here we aim merely at a description of the studies for the purpose of illustrating the role of experiments and potential causal claims in forensic methods. As we move on in subsequent sections there will be evaluative claims about these studies, but they concern the broader epistemological outlook of the studies’ motivation and communication – not the individual methodological choices and physiological assumptions.

Experimental asphyxia studies have been performed from the late 1980s (Reay et al., 1988) to the 2010s (Barnett et al., 2013) and continue to be discussed in the current medical literature (e.g., Steinberg, 2021; Vilke et al., 2022) both in forensic medicine and emergency medicine. The first experimental study of this kind was performed by Reay et al. (1988). The objective of the study was to examine the causal role of restraint in sudden or unexpected death-in-custody. More precisely, the study addressed the effects of positional restraint on peripheral oxygen saturation and heart rate. In the study 10 adult subjects first performed some mild exercise and were then placed in the PMRP. During exercise peripheral oxygen saturation and heart rate were monitored. The study found that the “effects of positional restraint produced a mean recovery that was significantly prolonged” (Reay et al., 1988, p. 16).

The study points out that the underlying mechanisms are unclear and could involve “restriction of thoracic respiratory movements, airway compromise, or physical stimulus of catecholamine release during exercise” (Reay et al., 1988, p. 18). What matters for our purposes here is what the authors of the study take these experiments to show for the real-world target phenomenon of death-in-custody. First, they note that the effects of a prolonged recovery could be exacerbated by other factors present in more realistic scenarios such as extreme physical exertion related to violent struggle, drug or ethanol intoxication or natural disease. Second, the study is careful with conclusions regarding actual field settings arguing that the experiment’s “relevance to the study of sudden and unexpected death remains unclear” and that more research is needed. Yet the main claim that “positional restraint and its effects should be considered when investigating deaths in persons who were handcuffed in the prone position” (ibid., 18) remains as the study’s key take-home message.

While the original study by Reay et al. suggests a potential causal relation between restraint and asphyxiation, there has been a series of experimental studies with a similar methodology that contests that causal relation. For example, Chan et al. (1997) performed a study that focused on whether restraint leads to clinically relevant respiratory dysfunction. More precisely, the study is motivated by the fact that “prehospital medical and law enforcement personnel often confront and care for violent, agitated individuals who must be subdued in order to prevent injury to themselves or others” (ibid., p. 579). So, again, the intended target is whether the studied restraint techniques can be seen as potential causes of death in struggle with police or medical personnel. Yet, here the more specific goal is to show that foregoing studies, e.g., by Reay et al. erroneously claim that the PMRP is harmful, that is, to show that the result of Reay et al. is a false positive. The study does find a restrictive pulmonary function pattern, but its main conclusion is that the restraint position “did not result in clinically relevant changes in oxygenation or ventilation” (ibid., p. 579). Still, the authors admit that it “is possible that a combination of factors, including underlying medical condition, intoxication, agitation, delirium, and struggle as well as body position, may result by respiratory compromise” (ibid., p. 585).

Subsequent studies have tested the effect of various factors that make the laboratory situation more akin to the target phenomenon in question. First, there is a series of studies that impose increasingly high weights on the test subjects placed in the PMRP (Chan et al., 2004; Michalewicz et al., 2007). For example, the study by Chan et al. (2004) looks at the “effect of 25 and 50 lbs weight force on respiratory function in human subject volunteers placed in the PMRP” and concludes that the restraint position with added weights “resulted in a restrictive pulmonary function pattern but no evidence of hypoxia or hypoventilation” (ibid., 185). Importantly also this study discusses some of the limitations of the applied methodology. It is pointed out that the laboratory study cannot reproduce potentially relevant aspects of the field setting such as “trauma, struggle, drug intoxication, and other physiologic and psychologic stresses that commonly occur with individuals who are being restrained in the field setting” (ibid., p. 188).

Moreover, there are studies looking at test subjects that have other risk factors. A study on obese subjects in PMRP reports that “there was no evidence of hypoxia or hypoventilation” (Sloane et al., 2014). By contrast, a study on subjects with chronic obstructive pulmonary disease (COPD) found that the effects of the PMRP were “highly individual, with some individuals tolerating the prone position with no measurable clinical effects and others suffering a clinical deterioration in symptoms” (Meredith et al., 2005, p. 133).

## Asphyxiation as potential cause of death

The initial Reay et al. study and, e.g., the study including COPD patients suggest that PMRP must be considered as a potential cause of asphyxia. Other studies contest this claim. Here we are not interested in deciding what claims are more plausible empirically. What matters for our purpose is what kind of claim is at stake in the first place.

The claim at stake in the reviewed experimental studies is whether the restraint position can cause asphyxia—a claim of potential causation. More specifically, the original study by Reay et al. can be seen as rebutting an impossibility claim. The tendency to put forward such claims is particularly evident from related discussions of the so-called Excited Delirium Syndrome (ExDS). ExDS is a controversial diagnosis, especially when invoked to explain death in custody (Fischer & Jukola, 2024). Among the problems is that there are no clear diagnostic criteria and that there arise questions as to whether ExDS is a genuine condition at all, with some arguing that its sole purpose is to explain deaths that are in fact related to excessive police violence.

A major argumentative strategy in support of the diagnosis is the lack of alternative explanations in presumed cases of death through ExDS. More precisely, it has been argued that the diagnosis often takes the form of a Holmesian inference: “(1) the fact that an individual died has an explanation; (2) the ExDS hypothesis and the asphyxia hypothesis are the only hypotheses that could explain the individual’s death; (3) the asphyxia hypothesis has been falsified; therefore (4) the ExDS hypothesis explains the individual’s death” (Fischer & Jukola, 2024, p. 41). The viability of this argumentative strategy crucially depends on (3), the claim that asphyxiation is not available as a competing explanation. Here impossibility claims regarding asphyxia come into play: only insofar as death by asphyxiation can be excluded, the ExDS hypothesis can be made plausible. This is even more important since the circumstantial evidence for both ExDS and asphyxia is typically highly elusive.

When it comes to evaluating claims of potential causation, we have argued that it is important to address both the range of the cause variable and the background conditions. First, consider range. This pertains to discussions of weights applied to test subjects as in the Chan et al. (2004) study. The study applies weights of 25 lbs and 50 lbs to the participants’ back and concludes that such weights do not make a relevant difference to the test subjects’ displaying signs of hypoxia or hypoventilation. Importantly, though, the study also points out that these weights may not reproduce the weights being applied in the field setting. The authors state that they “chose weight amounts which we felt would approximate weight force used in the field setting, heavy enough to indicate any trends if respiratory function was impacted, but not so heavy as to potentially place our subjects at risk of injury” (ibid., 188). Whether such “trends” continue to hold for weights applied in the field setting, however, is exactly the question that is at stake when claims of potential causation are evaluated with regard to the range of the causal variable. While later studies significantly increase applied weights, there remains the issue of whether applying such weight under controlled lab conditions amounts to the same forces encountered in violent struggle in the field setting.

Second, consider background conditions. It is well-accepted among both proponents and opponents of the death-by-asphyxiation hypothesis that cause of death in the relevant cases is often multifactorial. While it is not spelled out very clearly what that means there is at least one understanding of ‘multifactorial’ that would render the experiments problematic: if the joint influence of causal factors is needed for the effect to occur. The basic idea would be that each of the factors is not a sufficient condition of death. For example, obesity and recreational drug use, physical exertion due to struggle with the police, or certain restraint techniques alone would not lead to death. Yet the combination of one or more of these factors with restraint could be problematic. Consequently, experimental studies that isolate just one of the factors would not find that they represent a risk. It is only the combination of these factors that cause death by asphyxiation.

A principled problem for asphyxia studies is that the studies cannot be performed under realistic conditions because test subjects must not be put at risk. The authors of the experimental studies are aware that their experiments do not reproduce realistic field conditions. However, in most experiments realistic conditions are not realized. Consider, for example, a free-fall experiment aimed at showing the mass-independence of the free fall. The experiment shows the intended regularity only if the fall is shielded against the effect of aerodynamic drag force. The intended regularity would be shown by performing the experiment in an absolute vacuum. This would be technically highly demanding, and the goal can be achieved without producing an absolute vacuum. By repeating the experiment at various air pressures one can show that objects with different drag coefficients (such as a feather and stone) would be accelerated equally in the limiting case of vacuum.

One could argue that restraint experiments invoke the same principle of extrapolation. They aim to show that taking a certain restraint position after mild physical exertion and with weights applied to the test person’s back that are known to be harmless. Yet this kind of extrapolation works only under the assumption that changes in relevant background conditions do not yield a qualitatively different result. But, for example, the highly individual reaction of COPD patients in PMRP appears to indicate just that.

The primary role of forensic medical findings is the post-hoc evaluation of individual cases (actual causation). More specifically, they feature in the *justification* for claims of actual causation. A claim of actual causation can only be valid if there aren’t any impossibility claims blocking it: if it is established that a certain restraint technique cannot cause asphyxia, then one cannot cite the technique in a causal explanation of the death and alternative causes such as drug use or ExDS must be cited. Ultimately, this has consequences for who is to be held liable for the individual’s death.

Note that there is an important asymmetry here between claims that permit certain factors as causes and claims that exclude causes. To support a ‘can cause’ claim it is sufficient to show that under specific circumstances (that is, somewhere in the considered range of the cause variable or for at least one set of relevant background conditions) there is a dependence between the supposed cause and effect. In case of the ‘cannot cause’ claim, however, one has to exclude all relevant circumstances (that is: the full range of the cause variable, and all relevant background conditions) to ensure that the exclusion holds. This is difficult, if not impossible, because (1) it may simply be unknown what all relevant circumstances are and (2) require a highly complex, if not impossible, study design to realize them all.^6^

This also affects the role of such claims in the justification of claims of actual causation. The ‘cannot’ claim will block any causal claim of the relevant kind, it is thus evidentially strong (especially considering that much of the reasoning involved in forensic medicine goes by way of excluding possibilities; see, for example, the use of the ExDS diagnosis). The ‘can’ claim, however, is much weaker from an evidential viewpoint. The claim will be necessary to establish a causal connection but typically it is far from sufficient: we need additional evidence to turn a claim of ‘can’ causation to a claim of actual causation.

## False advertising

So far, we have discussed experimental work on asphyxia as an example of experiments that aim to establish or challenge claims of potential causation. Up to now, we have also avoided claims regarding the evaluation of these studies. In what follows we will shift gears and make normative claims. These claims, however, do not concern the methodology or internal validity of the studies. We leave such discussions to forensic medical experts. Instead, we will be concerned with specific challenges that arise from targeting and communicating claims of potential causation.

The problems we see with studies opposing the dangers of PMRP are related to what has been identified as “false advertising”: the “studies in question do not deal with the issues they purport to address” (Carrier, 2013, p. 2560). Intentions are usually impossible to show, and proving what the aspirations of involved individuals were is not necessary for labelling a case as an instance of false advertising (Carrier, 2020, p. 81f). What is relevant is “the unacknowledged difference between the questions to which a study is sensitive and those that are allegedly answered on its basis” (ibid, p. 82). In the case under consideration the issue the studies pretend to address is death by asphyxiation in the context of law enforcement officers and prehospital health personnel applying restraint techniques. The stated goal is to exclude that the restraint techniques are harmful. The methodology, however, is tuned to excluding a potentially wrong assumption that the restraint techniques are harmless.

Let us unpack this. The experimental studies by Chan et al. (2004) and studies with a similar design are instances of false advertising because of a mismatch between methodological design and their main claims: (1) they are aimed at excluding false positives, but (2) their design is not geared towards that aim. If at all, the study design would support the exclusion of false negatives. (1) The studies by Chan et al. and Schmidt and Snowden are performed in response to previous studies that indicated that the PMRP can be harmful in field cases. The stated concern of the asphyxia studies by Chan et al. and Schmidt and Snowden is primarily to exclude supposed *false positives*: the supposedly erroneous claim that certain restraint techniques are harmful. (2) However, the methodology of the studies is geared towards the exclusion of false negatives: the false assumption that certain restraint techniques are harmless when they are in fact problematic. If the laboratory studies can provide evidence for anything at all, then it is the claim that certain restraint positions *can* be harmful. The study, however, is not aptly designed to provide evidence that restraint positions *cannot* be harmful.

This last point is related to the noted asymmetry between ‘can cause’ claims and ‘cannot cause’ claims. To support a ‘can cause’ claim it is sufficient to show that under specific circumstances there is a dependence between the supposed cause and effect. This is what the studies can achieve: they perform specific instances of restraint under highly controlled conditions. If these instances indicate that the technique has a harmful effect on the test subject that would potentially allow an inference to the effect that PMRP-related asphyxiation can be a cause of death in the field setting. In case of the ‘cannot cause’ claim, however, one has to exclude all relevant scenarios to ensure that the exclusion holds. This is beyond the scope of the study design because of the various other factors that may come up in the field setting such as drug intoxication, factors that are explicitly acknowledged by the authors of these studies.

Note that these issues of false advertising do not affect the initial study of Reay et al. and similar studies. For these studies the stated aim and methods stand in a coherent relation. The studies are aimed at excluding false negatives: the potentially false assumption that restraint techniques are harmless in the field setting, and by providing evidence that restraint positions can cause asphyxiation they address exactly that assumption.

Mismatches between methodological design and claims are widely discussed in the sciences (Steel, 2018; Yarkoni, 2022). Moreover, such mismatches can have various reasons. In particular, they may result simply from methodological shortcomings or from intended misrepresentations. No matter what kind the current case is, the mismatches are particularly worrisome. As pointed out above, forensic medical experts often need to reason by way of excluding possibilities, and a main line of support for the problematic diagnosis of ExDS in death-in-custody cases has been the absence of alternative explanations. In this context, cannot-cause claims such as those put forward by the Chan et al. study are easily exploited and *have been* exploited to promote the case for ExDS, such as by DiMaio and DiMaio (2006). This means that false advertising, whether it is intentional or not, has serious ethical implications.

## Conclusion and outlook

Our focus has been the peculiar role that experiments performed under controlled laboratory conditions play in the assessment of highly individual causal claims. We have argued that the main purpose of experimentation in forensic medicine is to establish claims of potential causation. These claims typically do not provide the evidential resources for explaining an individual’s death. Yet they constitute an important guideline for the search of an individual’s actual cause of death. More specifically, experiments in forensic medicine are often concerned with the rebuttal of impossibility claims. When it is in principle impossible that a certain kind of circumstance causes an individual’s death, then this lends support to alternative explanations. We have also pointed out that such claims of potential causation are affected by a number of distinct epistemic difficulties. In particular, one needs to specify the relevant range and background conditions of such claims in order to establish them empirically.

We have illustrated this on the example of experimental studies on the restraint positions used by law enforcement. While some studies identify these as potential causes of asphyxiation, others have argued no such conclusions can be drawn from experiments. Here we did not provide an assessment of the empirical studies themselves. Instead, we have used them to make clear that what matters in such experimental studies are claims of potential causation. A general lesson to be drawn from this look into asphyxiation studies is that issues of false advertising threaten the communication of empirical results about potential causation. The issue that asphyxia studies pretend to address is death by asphyxiation in the context of law enforcement officers and prehospital health personnel applying restraint techniques. The stated goal of some of these studies is to exclude that the restraint techniques are harmful. The methodology, however, is tuned to excluding a supposedly false assumption that the restraint techniques are harmless.

The claims made on the example of asphyxiation studies extend to other instances of experimentation in forensic medicine. Consider experimental studies on wound ballistics (Coupland 2011; Carr et al., 2018). These studies do not provide evidence regarding the actual cause of an individual’s gunshot wound, but merely evidence regarding potential causes, by examining various factors such as bullet type, body armor, and body position that can have an impact on wound morphology. Moreover, issues of ‘can’ vs. ‘cannot cause’ similar to those encountered in the discussion of asphyxiation apply here, for instance, when projectiles are optimized for survivability, or when weapons need to be admitted for use by law enforcement and medical personnel (see, e.g., research on Conducted Electrical Weapons, such as TASER^®^, e.g., Kunz et al., 2018).

Let us conclude with two overarching considerations. First, experiments in forensic medicine represent a distinct mode of creating evidential support for concrete cause-of-death hypotheses. There is an overarching question as to how this kind of evidence is to be seen in relation to other forms of evidence commonly employed in forensic medicine, especially through the use of case studies. While some argue that experimental work is generally more reliable than evidence from case reports,^7^ the picture suggested by our analysis of experiments is a different one. Experiments merely provide the basis for claims of potential causation. How these are to be applied to specific cases depends on concrete background conditions of the case. How these are to be evaluated, is a separate question that may be fruitfully addressed by invoking case reports that can “serve as ‘vehicles’ for gathering facts and putting them into contact with each other” (Ankeny, 2014, p. 1000).

Second, the causal claims that are at stake in forensic medicine do not just concern the post-hoc evaluation of individual cases. Another important role of the causal claims examined here is policy recommendation: what kinds of methods should be applied to control individuals in custody or in prehospital situations? Should certain restraint techniques be banned or should law enforcement officers be allowed or even be taught and encouraged to employ them? Such are questions of concrete decision-making that will depend on a variety of additional issues such as the availability of alternative methods and how the risks associated with these alternatives are to be evaluated. The discussed epistemic asymmetry between ‘can cause’ and ‘cannot cause’ as well as related issues of false advertising have additional relevance when it comes to such decisions. Concretely, the kind of false advertising we have highlighted here bears the risk of being employed to justify potentially harmful policies.

## Data Availability

Not applicable.

## Footnotes

1 Further issues that have received epistemological attention are questions of objectivity (Koolage et al., 2021), replicability (Sikorski, 2022), and expert testimony (Peruzzi, 2023).

2 Questions about the validity of the outcomes of experiments and trials are of course not limited to forensic medicine. In medical research, the problem of external validity concerns also RCTs and has been discussed by both medical researchers and philosophers (Rothwell, 2005; Solomon, 2011; Fuller, 2021).

3 There is an ongoing debate regarding what “bring about” means, both in the philosophy of causation (e.g., Lewis, 1973; Hitchcock, 2001; Halpern & Pearl, 2005; Beckers & Vennekens, 2018; Andreas & Günther, 2021; Fischer, 2024b) and the legal literature (e.g., Wright, 1985; Moore, 2009). For example, a straightforward counterfactual analysis (or ‘but-for test’) of actual causation tends to be too restrictive in cases involving causal overdetermination. When two individually lethal bullets hit a victim’s body they are typically identified as actual causes, even though the victim’s death does not depend counterfactually on either bullet. Another potential problem is that not all factors that an event depends on are usually identified as actual causes. For example, the victim’s death is thought to be caused by the shooter pulling the trigger of their gun but not by the shooter’s birth. While such cases raise exciting issues for the philosophy of causation and will have interesting consequences for some forensic cases, they are not relevant for the following analysis.

4 See Woodward’s (2003) notion of “contributing cause” for a similar concept and delineation from actual causation. For a more detailed discussion of the differences between actual and potential causation see Fischer (2021a).

5 The issue described here as context-sensitivity is parallel, for example, to well-known issues of variable relativity discussed in the causal modelling literature (Woodward, 2016; Statham, 2018; Parkkinen, 2022; Fischer, 2021b, 2024a). To assess causal relations in the causal modelling framework one has to first make a decision about the possible values of the variables that are part of the causal model (this corresponds to the issue of background conditions) and the possible values of the variables (this corresponds to the issue of range).

6 As an analogy consider pharmacology. As has been argued by, e.g., Osimani (2013), RCTs are not a good method for gaining evidence of the unexpected adverse effects of interventions or their absence. Thanks to an anonymous reviewer for pointing this out.

7 Proponents of the studies finding no relation between restraint positions and asphyxiation have seen criticism based on case reports. In this context its proponents have defended experimental studies as a “lovely piece of pathology research” that is challenged by “what today could only be described as junk science” (Karch et al., 2016, p. 75).
